# Bis(2-amino­thia­zolium) succinate succinic acid

**DOI:** 10.1107/S1600536809007004

**Published:** 2009-03-14

**Authors:** Hoong-Kun Fun, Jain John, Samuel Robinson Jebas, T. Balasubramanian

**Affiliations:** a X-ray Crystallography Unit, School of Physics, Universiti Sains Malaysia, 11800 USM, Penang, Malaysia; b Department of Physics, National Institute of Technology, Tiruchirappalli 620 015, India

## Abstract

In the title compound, 2C_3_H_5_N_2_S^+^·C_4_H_4_O_4_^2−^·C_4_H_6_O_4_, the thia­zolium ring is almost planar, with the maximum deviation from planarity being 0.0056 (8) Å for the C atom carrying the amine substituent. The N atom of the 2-amino­thia­zole mol­ecule is protonated. Both the anion and the acid lie across inversion centres. The crystal packing is consolidated by inter­molecular O—H⋯O, N—H⋯O and C—H⋯O hydrogen bonds. Mol­ecules are stacked down the *b* axis.

## Related literature

For the structure of 2-amino­thia­zole, see: Caranoni & Reboul (1982 ▶). For applications of 2-amino­thia­zole, see: Saarnivaara & Matilla (1974 ▶); Windholz (2001 ▶). For the structure of succinic acid, see: Gopalan *et al.* (2000 ▶); Leviel *et al.* (1981 ▶). For applications of succinic acid, see: Sauer *et al.* (2008 ▶); Song & Lee (2006 ▶); Zeikus *et al.* (1999 ▶). For the stability of the temperature controller used in the data collection, see: Cosier & Glazer (1986 ▶). 
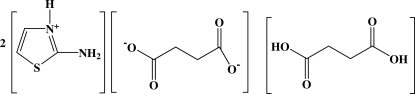


## Experimental

### 

#### Crystal data

2C_3_H_5_N_2_S^+^·C_4_H_4_O_4_^2−^·C_4_H_6_O_4_
*M* *_r_* = 436.46
Monoclinic, 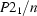

*a* = 10.1680 (2) Å
*b* = 5.1012 (1) Å
*c* = 18.3850 (4) Å
β = 105.961 (1)°
*V* = 916.85 (3) Å^3^
*Z* = 2
Mo *K*α radiation
μ = 0.34 mm^−1^
*T* = 100 K
0.58 × 0.42 × 0.32 mm

#### Data collection

Bruker SMART APEXII CCD area-detector diffractometer
Absorption correction: multi-scan (*SADABS*; Bruker, 2005 ▶) *T*_min_ = 0.826, *T*_max_ = 0.897
16689 measured reflections
3691 independent reflections
3442 reflections with *I* > 2σ(*I*)
*R*_int_ = 0.022

#### Refinement

*R*[*F* ^2^ > 2σ(*F* ^2^)] = 0.027
*wR*(*F* ^2^) = 0.077
*S* = 1.04
3691 reflections
167 parameters
All H-atom parameters refined
Δρ_max_ = 0.45 e Å^−3^
Δρ_min_ = −0.35 e Å^−3^

### 

Data collection: *APEX2* (Bruker, 2005 ▶); cell refinement: *SAINT* (Bruker, 2005 ▶); data reduction: *SAINT*; program(s) used to solve structure: *SHELXTL* (Sheldrick, 2008 ▶); program(s) used to refine structure: *SHELXTL*; molecular graphics: *SHELXTL*; software used to prepare material for publication: *SHELXTL* and *PLATON* (Spek, 2009 ▶).

## Supplementary Material

Crystal structure: contains datablocks global, I. DOI: 10.1107/S1600536809007004/sj2582sup1.cif

Structure factors: contains datablocks I. DOI: 10.1107/S1600536809007004/sj2582Isup2.hkl

Additional supplementary materials:  crystallographic information; 3D view; checkCIF report

## Figures and Tables

**Table 1:** Hydrogen-bond geometry (Å, °)

*D*—H⋯*A*	*D*—H	H⋯*A*	*D*⋯*A*	*D*—H⋯*A*
N2—H1*N*2⋯O1^i^	0.868 (15)	1.974 (15)	2.8156 (9)	163.0 (14)
N2—H2*N*2⋯O1^ii^	0.889 (14)	1.959 (13)	2.8297 (9)	165.7 (13)
N1—H1*N*1⋯O2^i^	0.865 (14)	1.823 (14)	2.6868 (8)	176.2 (15)
O3—H1*O*3⋯O2^ii^	0.888 (19)	1.696 (19)	2.5820 (8)	176.5 (19)
C1—H1⋯O4^iii^	0.985 (13)	2.431 (14)	3.3086 (10)	148.2 (12)

Symmetry codes: (i) 
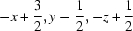
; (ii) 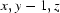; (iii) 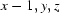.

**Fig. 1.**
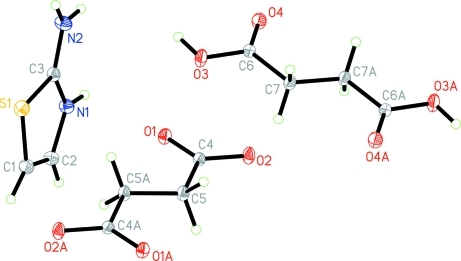
The molecular structure of the title compound, showing 50% probability displacement ellipsoids and the atom numbering scheme. [symmetry operators used to generate equivalent atoms are (i) -x + 1, -y + 2, -z and (ii) -x + 2, -y + 2, -z for the anion and acid respectively].

**Fig. 2.**
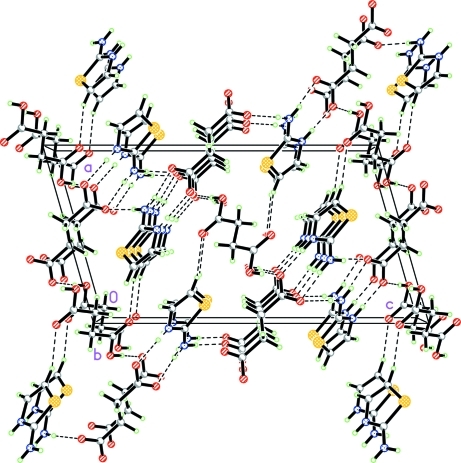
The crystal packing of the title compound, viewed along the b axis. Dashed lines indicate the hydrogen bonding.

## supplementary crystallographic information

### Comment

2-Aminothiazole derivatives have shown heribicidal, anti-inflammatory, anti-microbial and antiparasitic activities (Saarnivaara & Matilla, 1974). 2-Aminothiazole is listed as a thyroid inhibitor (Windholz, 2001). The orthorhombic form of 2-amino-1,3-thiazole has been reported (Caranoni & Reboul, 1982). Succinic acid is a dicarboxylic acid and is a precursor for many chemicals of industrial importance (Zeikus *et al.*, 1999; Song & Lee, 2006). Succinic acid derivatives are mostly used in chemicals, food and pharmaceuticals (Sauer *et al.*, 2008). The crystal structure of succinic acid has been reported (Gopalan *et al.*, 2000; Leviel *et al.* 1981). Due to all these important properties, the title compound (I) has been synthesized and is reported here.

The asymmetric unit of (I) (Fig. 1) contains one molecule of protonated 2-aminothiazole, a half molecule of succinate and a half molecule of succinic acid. The anion and the acid lie across different inversion centres [symmetry codes (i) -*x* + 1, -*y* + 2, -*z* and (ii) -*x* + 2, -*y* + 2, -*z* respectively]. The thiazolium ring is planar with the maximum deviation from planarity being 0.0056 (8)Å for atom C3. The ring nitrogen of the 2-aminothiazole molecule is protonated thereby leading to a widening of the corresponding internal angle (C2–N1–C3) of the thiazolium ring to 113.99 (6)° [which is 109.4 (5)° in the unprotonated form of 2-aminothiazole] and an increase in the bond lengths of N1–C3 to 1.334 (9)Å and N1–C2 to 1.387 (9)Å [which are 1.298 (6)Å and 1.375 (6)Å respectively in its uncomplexed form] (Caranoni & Reboul, 1982). An increase in the C1–S1–C3 bond angle to 90.47 (3)° [which is 88.6 (3)° in the unprotonated form] and a decrease in the S1–C3–N1 bond angle to 111.38 (5)° [which is 114.9 (5)° in the uncomplexed form of 2-aminothiazole] (Caranoni & Reboul, 1982) are also observed.

The bond lengths and bond angles of the succinate and succinic acid are found to have normal values (Gopalan *et al.*, 2000; Leviel & Auvert, 1981). The crystal packing is consolidated by O—H···O, N—H···O and C—H···O intermolecular hydrogen bonds (Table 1) together with intermolecular [O—O^i^ = 2.5820 (8) Å; O—N^ii-iii^ = 2.6868 (8) to 2.8297 (9) Å, short contacts [symmetry code: (i) *x*,-1 + *y*,*z*; (ii) 3/2 - *x*,1/2 + *y*,1/2 - *z*; (iii) *x*,1 + *y*,*z*]. Molecules are stacked down the *b* axis(Fig.2).

### Experimental

2- Aminothiazole (0.100 g, 1 mmol) and succinic acid (0.118 g, 1 mmol) were dissolved in ethanol (25 ml) in a 1:1 molar ratio. The clear brown solution obtained was refluxed for 6 h at a temperature of 323 K. Brown coloured crystals were harvested after two weeks on slow evaporation of the solvent.

### Refinement

All the hydrogen atoms were located from the Fourier map and were allowed to refine freely with isotropic displacement parameters.

### Crystal data

**Table:**

2C_3_H_5_N_2_S^+^·C_4_H_4_O_4_^2^^−^·C_4_H_6_O_4_	*F*(000) = 456
*M**_r_* = 436.46	*D*_x_ = 1.581 Mg m^−^^3^
Monoclinic, *P*2_1_/*n*	Mo *K*α radiation, λ = 0.71073 Å
Hall symbol: -P 2yn	Cell parameters from 9994 reflections
*a* = 10.1680 (2) Å	θ = 2.3–40.1°
*b* = 5.1012 (1) Å	µ = 0.34 mm^−^^1^
*c* = 18.3850 (4) Å	*T* = 100 K
β = 105.961 (1)°	Block, yellow
*V* = 916.85 (3) Å^3^	0.58 × 0.42 × 0.32 mm
*Z* = 2	

### Data collection

**Table:**

Bruker SMART APEXII CCD area-detector diffractometer	3691 independent reflections
Radiation source: fine-focus sealed tube	3442 reflections with *I* > 2σ(*I*)
graphite	*R*_int_ = 0.022
φ and ω scans	θ_max_ = 34.0°, θ_min_ = 2.1°
Absorption correction: multi-scan (*SADABS*; Bruker, 2005)	*h* = −16→16
*T*_min_ = 0.826, *T*_max_ = 0.897	*k* = −7→7
16689 measured reflections	*l* = −28→24

### Refinement

**Table:**

Refinement on *F*^2^	Primary atom site location: structure-invariant direct methods
Least-squares matrix: full	Secondary atom site location: difference Fourier map
*R*[*F*^2^ > 2σ(*F*^2^)] = 0.027	Hydrogen site location: difference Fourier map
*wR*(*F*^2^) = 0.077	All H-atom parameters refined
*S* = 1.04	*w* = 1/[σ^2^(*F*_o_^2^) + (0.0423*P*)^2^ + 0.2556*P*] where *P* = (*F*_o_^2^ + 2*F*_c_^2^)/3
3691 reflections	(Δ/σ)_max_ = 0.001
167 parameters	Δρ_max_ = 0.45 e Å^−^^3^
0 restraints	Δρ_min_ = −0.35 e Å^−^^3^

### Special details

**Table:**

Experimental. The crystal was placed in the cold stream of an Oxford Cyrosystems Cobra open-flow nitrogen cryostat (Cosier & Glazer, 1986) operating at 100.0 (1) K.
Geometry. All e.s.d.'s (except the e.s.d. in the dihedral angle between two l.s. planes) are estimated using the full covariance matrix. The cell e.s.d.'s are taken into account individually in the estimation of e.s.d.'s in distances, angles and torsion angles; correlations between e.s.d.'s in cell parameters are only used when they are defined by crystal symmetry. An approximate (isotropic) treatment of cell e.s.d.'s is used for estimating e.s.d.'s involving l.s. planes.
Refinement. Refinement of *F*^2^ against ALL reflections. The weighted *R*-factor *wR* and goodness of fit *S* are based on *F*^2^, conventional *R*-factors *R* are based on *F*, with *F* set to zero for negative *F*^2^. The threshold expression of *F*^2^ > σ(*F*^2^) is used only for calculating *R*-factors(gt) *etc*. and is not relevant to the choice of reflections for refinement. *R*-factors based on *F*^2^ are statistically about twice as large as those based on *F*, and *R*- factors based on ALL data will be even larger.

### Fractional atomic coordinates and isotropic or equivalent isotropic displacement parameters (Å^2^)

**Table:**

	*x*	*y*	*z*	*U*_iso_*/*U*_eq_	
S1	0.398521 (18)	0.38496 (4)	0.154349 (10)	0.01603 (6)	
O1	0.64045 (5)	0.89655 (11)	0.12963 (3)	0.01367 (10)	
O2	0.77343 (5)	1.19344 (12)	0.09559 (3)	0.01562 (10)	
O3	0.80012 (6)	0.57645 (12)	0.00894 (3)	0.01703 (11)	
O4	0.99583 (6)	0.66022 (12)	0.09808 (3)	0.01646 (11)	
N1	0.52407 (6)	0.60443 (12)	0.27794 (3)	0.01361 (11)	
N2	0.63526 (6)	0.22575 (14)	0.25269 (4)	0.01565 (12)	
C1	0.32820 (7)	0.66562 (16)	0.18253 (4)	0.01749 (13)	
C2	0.40776 (7)	0.75502 (15)	0.24905 (4)	0.01599 (13)	
C3	0.53433 (7)	0.39842 (14)	0.23509 (4)	0.01261 (12)	
C4	0.66461 (6)	1.05440 (14)	0.08264 (4)	0.01101 (11)	
C5	0.56100 (7)	1.09079 (14)	0.00653 (4)	0.01307 (12)	
C6	0.91584 (7)	0.70771 (13)	0.03682 (4)	0.01173 (11)	
C7	0.93722 (7)	0.91684 (14)	−0.01673 (4)	0.01291 (12)	
H1	0.2400 (13)	0.731 (3)	0.1506 (7)	0.027 (3)*	
H2	0.3948 (13)	0.900 (2)	0.2779 (7)	0.023 (3)*	
H7A	0.8557 (13)	1.025 (3)	−0.0314 (7)	0.023 (3)*	
H7B	0.9449 (13)	0.826 (3)	−0.0630 (7)	0.025 (3)*	
H1N2	0.7022 (15)	0.248 (3)	0.2933 (8)	0.035 (4)*	
H2N2	0.6384 (13)	0.100 (3)	0.2197 (7)	0.025 (3)*	
H1C5	0.6093 (14)	1.070 (3)	−0.0333 (8)	0.030 (3)*	
H2C5	0.5330 (13)	1.280 (3)	0.0047 (8)	0.030 (3)*	
H1N1	0.5877 (14)	0.640 (3)	0.3188 (8)	0.031 (3)*	
H1O3	0.7940 (17)	0.447 (4)	0.0401 (9)	0.052 (5)*	

### Atomic displacement parameters (Å^2^)

**Table:**

	*U*^11^	*U*^22^	*U*^33^	*U*^12^	*U*^13^	*U*^23^
S1	0.01505 (8)	0.01672 (9)	0.01227 (8)	−0.00239 (5)	−0.00303 (6)	0.00025 (5)
O1	0.0141 (2)	0.0152 (2)	0.0103 (2)	−0.00308 (17)	0.00095 (16)	0.00264 (16)
O2	0.0127 (2)	0.0175 (2)	0.0136 (2)	−0.00582 (18)	−0.00159 (17)	0.00353 (18)
O3	0.0150 (2)	0.0181 (3)	0.0151 (2)	−0.00693 (18)	−0.00061 (18)	0.00485 (19)
O4	0.0145 (2)	0.0178 (2)	0.0146 (2)	−0.00236 (18)	−0.00011 (18)	0.00380 (19)
N1	0.0128 (2)	0.0147 (3)	0.0116 (2)	0.00017 (19)	0.00047 (19)	−0.00062 (19)
N2	0.0141 (2)	0.0176 (3)	0.0128 (2)	0.0022 (2)	−0.0003 (2)	−0.0030 (2)
C1	0.0137 (3)	0.0171 (3)	0.0189 (3)	−0.0001 (2)	−0.0001 (2)	0.0044 (3)
C2	0.0141 (3)	0.0152 (3)	0.0179 (3)	0.0014 (2)	0.0031 (2)	0.0022 (2)
C3	0.0119 (3)	0.0143 (3)	0.0102 (2)	−0.0016 (2)	0.0008 (2)	0.0004 (2)
C4	0.0106 (2)	0.0116 (3)	0.0097 (2)	−0.0009 (2)	0.00098 (19)	0.0002 (2)
C5	0.0115 (2)	0.0153 (3)	0.0101 (2)	−0.0035 (2)	−0.0009 (2)	0.0027 (2)
C6	0.0113 (2)	0.0111 (3)	0.0127 (3)	−0.0006 (2)	0.0032 (2)	0.0002 (2)
C7	0.0129 (3)	0.0129 (3)	0.0122 (3)	−0.0022 (2)	0.0023 (2)	0.0018 (2)

### Geometric parameters (Å, °)

**Table:**

S1—C3	1.7285 (7)	N2—H2N2	0.888 (13)
S1—C1	1.7416 (9)	C1—C2	1.3468 (11)
O1—C4	1.2538 (8)	C1—H1	0.986 (13)
O2—C4	1.2802 (8)	C2—H2	0.941 (13)
O3—C6	1.3281 (8)	C4—C5	1.5139 (9)
O3—H1O3	0.887 (18)	C5—C5^i^	1.5135 (14)
O4—C6	1.2185 (8)	C5—H1C5	0.993 (14)
N1—C3	1.3346 (9)	C5—H2C5	1.003 (14)
N1—C2	1.3877 (9)	C6—C7	1.5075 (10)
N1—H1N1	0.864 (14)	C7—C7^ii^	1.5153 (14)
N2—C3	1.3233 (9)	C7—H7A	0.970 (13)
N2—H1N2	0.868 (15)	C7—H7B	0.989 (13)
			
C3—S1—C1	90.47 (3)	O1—C4—C5	119.82 (6)
C6—O3—H1O3	109.7 (10)	O2—C4—C5	116.75 (6)
C3—N1—C2	113.99 (6)	C5^i^—C5—C4	113.78 (7)
C3—N1—H1N1	121.0 (9)	C5^i^—C5—H1C5	111.6 (8)
C2—N1—H1N1	125.0 (9)	C4—C5—H1C5	108.0 (8)
C3—N2—H1N2	119.6 (10)	C5^i^—C5—H2C5	111.7 (8)
C3—N2—H2N2	118.9 (8)	C4—C5—H2C5	105.5 (8)
H1N2—N2—H2N2	121.0 (13)	H1C5—C5—H2C5	105.7 (11)
C2—C1—S1	110.81 (6)	O4—C6—O3	123.50 (6)
C2—C1—H1	130.3 (8)	O4—C6—C7	124.39 (6)
S1—C1—H1	118.9 (8)	O3—C6—C7	112.10 (6)
C1—C2—N1	113.33 (7)	C6—C7—C7^ii^	112.82 (7)
C1—C2—H2	129.5 (8)	C6—C7—H7A	108.6 (7)
N1—C2—H2	117.2 (8)	C7^ii^—C7—H7A	110.8 (8)
N2—C3—N1	124.12 (6)	C6—C7—H7B	106.9 (8)
N2—C3—S1	124.49 (5)	C7^ii^—C7—H7B	110.7 (7)
N1—C3—S1	111.38 (5)	H7A—C7—H7B	106.8 (10)
O1—C4—O2	123.42 (6)		
			
C3—S1—C1—C2	0.33 (6)	C1—S1—C3—N1	−0.78 (6)
S1—C1—C2—N1	0.18 (9)	O1—C4—C5—C5^i^	−4.29 (11)
C3—N1—C2—C1	−0.80 (9)	O2—C4—C5—C5^i^	176.67 (8)
C2—N1—C3—N2	−178.46 (7)	O4—C6—C7—C7^ii^	−5.84 (12)
C2—N1—C3—S1	1.04 (8)	O3—C6—C7—C7^ii^	175.31 (7)
C1—S1—C3—N2	178.72 (7)		

Symmetry codes: (i) −*x*+1, −*y*+2, −*z*; (ii) −*x*+2, −*y*+2, −*z*.

### Hydrogen-bond geometry (Å, °)

**Table:**

*D*—H···*A*	*D*—H	H···*A*	*D*···*A*	*D*—H···*A*
N2—H1N2···O1^iii^	0.868 (15)	1.974 (15)	2.8156 (9)	163.0 (14)
N2—H2N2···O1^iv^	0.889 (14)	1.959 (13)	2.8297 (9)	165.7 (13)
N1—H1N1···O2^iii^	0.865 (14)	1.823 (14)	2.6868 (8)	176.2 (15)
O3—H1O3···O2^iv^	0.888 (19)	1.696 (19)	2.5820 (8)	176.5 (19)
C1—H1···O4^v^	0.985 (13)	2.431 (14)	3.3086 (10)	148.2 (12)

Symmetry codes: (iii) −*x*+3/2, *y*−1/2, −*z*+1/2; (iv) *x*, *y*−1, *z*; (v) *x*−1, *y*, *z*.
